# Precise phase demodulation of single carrier-frequency interferogram by pixel-level Lissajous figure and ellipse fitting

**DOI:** 10.1038/s41598-017-18031-4

**Published:** 2018-01-09

**Authors:** Fengwei Liu, Yongqian Wu, Fan Wu, Niels König, Robert Schmitt, Yongjian Wan, Yan Xu

**Affiliations:** 1 The Institute of Optics and Electronics, Chinese Academy of Sciences, Chengdu, Sichuan 610209 China; 2 University of Chinese Academy of Sciences, Beijing, 100039 China; 3 Fraunhofer Institute for Production technology IPT, 52074 Aachen, Germany; 4 Laboratory for Machine Tools and Production Engineering (WZL) of RWTH Aachen University, 52074 Aachen, Germany

## Abstract

Phase demodulation from a single carrier-frequency fringe pattern is becoming increasingly important particularly in areas of optical metrology such as dynamic interferometry, deflectometry and profilometry. The Fourier transform (FT) method and the spatial-carrier phase-shifting technique (SCPS) are two popular and well-established approaches to demodulation. However FT has the drawback of significant edge errors because of the Gibbs effect, whilst detuning errors for the local phase shift occur when SCPS is applied. A novel demodulation method based on pixel-level Lissajous figure and ellipse fitting (PLEF) is presented in this paper. Local demodulation in the spatial domain makes PLEF more flexible than the FT method, without spectral leakage. Based on a more adaptable approach, account is taken of variations in illumination and phase distribution over a few neighboring pixels. The mathematic demodulation model is of interest and has been demonstrated via simulation. Theoretical phase extraction error is as low as 10^−4^ rad. Experiments further corroborate the effectiveness of the proposed method. In conclusion, various influencing factors, e.g. variations of background/modulation, phase amplitude, carrier frequency, additive noise that may affect the precision of PLEF are discussed in detail.

## Introduction

Interferometry, deflectometry and profilometry are diagnostic tools frequently used in optical metrology to achieve highly accurate, non-contact and full-field measurements. Since the relevant physical information is usually encoded in the fringe phase, phase demodulation always plays a very critical role in fringe analysis. Temporal phase shifting (TPS) is a phase demodulation method, which has been used extensively in optical metrology since its introduction^1^.

Phase shifting interferometry (PSI), for example, has already become a standard configuration of a modern high accuracy interferometer^2^. A well-calibrated phase shifter and stable environment are crucial factors to TPS due to equal steps conventionally used in phase-shifting algorithms (PSA)^3,4^. Though various kinds of generalized PSI have been put forward in recent years as a means of coping with random phase shift error caused by vibration^5–9^, it remains difficult to achieve real time phase demodulation in dynamic measurement operations since at least three interferograms are usually required in order to record and process data. Dynamic measurement is an attractive option not only in optical shop testing, e.g. high-aperture and long-distance primary mirror testing^10^ but also in many other phase-demodulation operations, e.g. visual monitoring of cell culture in the biomedical domain^11^. With the exception of simultaneous phase shifting interferometry (SPSI)^12^, which is not our concern in this contribution, demodulation of a single shot fringe pattern is an attractive option. The spatial carrier-frequency (SCF) method is a good choice since implementation of spatial carrier-frequency interferometry (SCFI) is relatively easily accomplished on most advanced laser interferometers. The retrace error is the main disadvantage of SCFI but can be calibrated out in advance. For a carrier-frequency interferogram (CFI), the FT method, first proposed in 1982 by Takeda M. *et al*.^13^ is a mainstay technique used to perform phase demodulation. Due to the global operation mechanism in the frequency domain, a lack of spatial localization might lead to spectral overlapping, rendering it almost impossible to extract the first-order component accurately^14^. The windowed FT(WFT) method^15^ was therefore suggested as a way of improving spatial localization, but the automatic selection of a window remains a major problem for dynamic measurement. The TPS technique is well known for single-point-level spatial localization, and it is possible to transfer the TPS concept to spatial CFI analysis, which is called the spatial carrier-frequency phase shifting (SCPS) technique^16^. It uses the phase increment of adjacent pixels in intra-frame to approximate the carrier frequency. Based on this approximation, which also assumes that the background intensities and modulation amplitudes of adjacent pixels are equal, the single CFI can be decomposed into a series of temporal phase shifted interferograms. Hereafter, the classic SCPS demodulates the phase using conventional PSAs with the predetermined carrier frequency of *π*/2 rad/pixel^17^. With the development of generalized PSA in temporal PSI, especially the advanced iterative algorithm (AIA)^7^ and the principle component analysis method (PCA)^18^, the CFI can now be demodulated without pre-calibrating the carrier frequency^19–22^. However, variations in background/modulation and local phase shift over adjacent pixels are still the main error sources leading to distinct detuning errors in all modified algorithms, except for the iterative least squares method (ILSM) proposed by Xu *et al*.^19^. Even though ILSM can compensate for variations in illumination and local phase shift between adjacent pixels, the iterative strategy requires considerable computation. Additionally, the convergent problem is tricky when the fringe tilt angle in the interferogram approximates zero or 90 degree. Though PCA-based SCPS^20^ can demodulate the CFI at very high speed, there is no compensation for local phase shift and the PCA itself is less accurate when both the number of interferograms and the phase shifts between them are small^23–26^. In terms of accuracy and speed, ILSM is sluggish, e.g. it costs 27.6 s if the size of CFI is 400 × 400^20^ and PCA-based SCPS, is less accurate, e.g. the demodulation error is 0.041 rad (RMS) even the noise level is zero; all have deficiencies in relation to the need for high accuracy dynamic measurement.

In our previously published works^9,27^, the Lissajous figure and ellipse-fitting (LEF) was shown to be effective in the correction of phase extraction errors in TPS and was successfully extended to a generalized PSA by combining with AIA. This differs from the PCA or other temporal PSA-based SCPS methods, which simply transfer the temporal PSA into SCPS to perform demodulation. In this report, we propose a pixel-level Lissajous figure and ellipse fitting (PLEF) method, which does not require the construction of four or nine temporal, phase-shifted interferograms. The CFI will be demodulated pixel by pixel, thereby permitting variations in background/modulation and local phase shift to be fully considered and well compensated. It also differs considerably from our previous LEF-based methods (ETC^27^, ETCI^9^) the pixel-level Lissajous figure here is created using nonuniform illumination points. The background/modulation and even the local phase shift at each pixel can be simultaneously demodulated. Using these parameters, the final encoded phase can be reconstructed in many different ways, e.g. it can be integrated with local phase shift (without phase unwrapping process)^21^, demodulated directly from two sinusoidal expressions^27^ and the least squares fitting method can also be applied^21,22^. Detailed characteristics and the capability of the method proposed in this paper will be discussed and corroborated in the following sections.

## Methods

It is well known that the Lissajous figure, typically an ellipse, can be expressed as two sinusoidal signals:

1 $$N(t)=a+b\cdot \,\sin [\phi (t)],\,D(t)=a+b\cdot \,\cos [\phi (t)+\delta ].$$

where *a* is the offset, *b* is the modulation and *δ* is the quadrature phase shift error. *φ*(*t*) is the encoded phase which varies with *t*. If the variable *t* modifies *φ*(*t*) over a whole phase period, i.e. 2*π*, then the Lissajous figure is closed. The offset *a*, modulation *b* and quadrature phase shift error *δ* can all be demodulated using an ellipse fitting algorithm (EFA). Note that the ellipse is only error free when *a*, *b* and *δ* stay the same while *t* varies, it is called the perfect condition (PC). The process is known as Lissajous figure and ellipse fitting (LEF) in literature^27–29^.

We also know the intensity of a CFI can be expressed mathematically as,

2 $$I(x,y)=A(x,y)+B(x,y)\cdot \,\cos \,[\phi (x,y)+{k}_{x}\cdot x+{k}_{y}\cdot y]$$

where *A*(*x*, *y*), *B*(*x*, *y*) represent the background intensity and the modulation amplitude of the fringe respectively, *x*, *y* denote the pixel coordinate. *φ*(*x*, *y*) is object phase, *k*_*x*_, *k*_*y*_ are the spatial carrier-frequencies along *x* and *y* directions.

**Figure 1:**
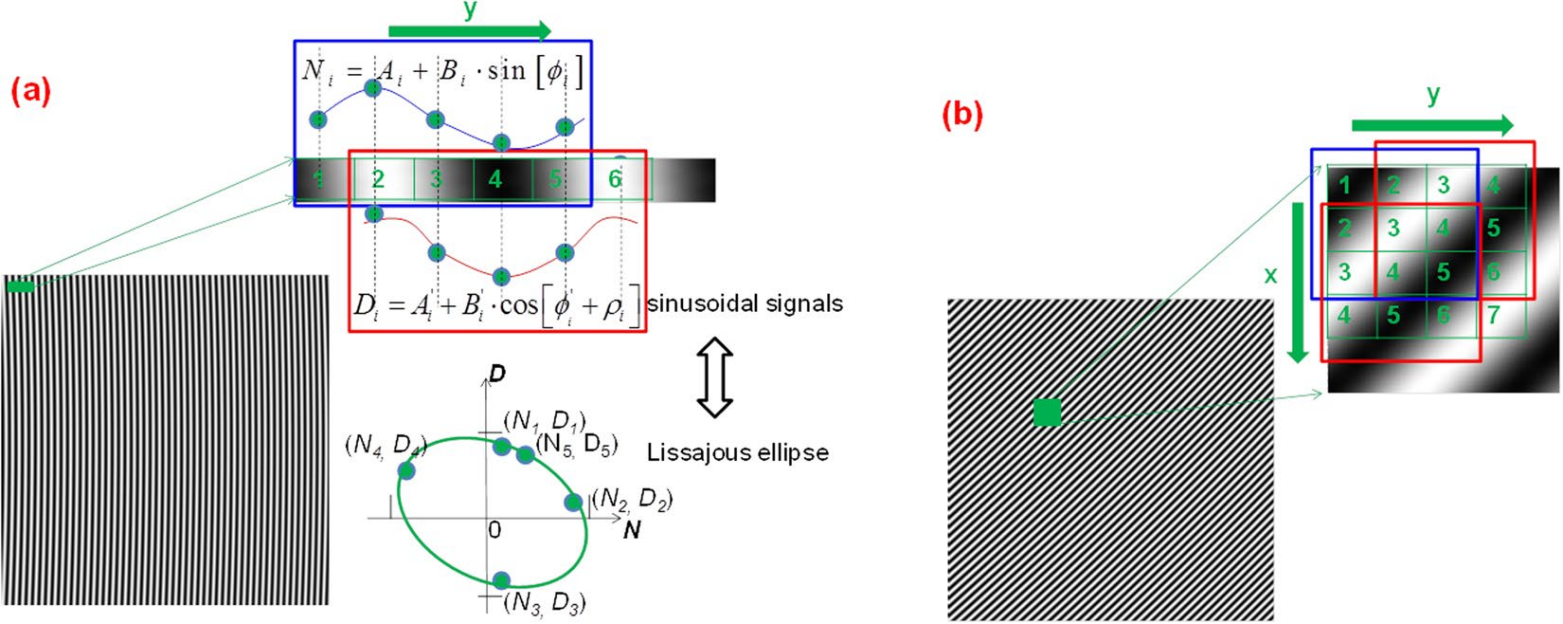
The 1D mode (**a**) and 2D mode (**b**) of producing Lissajous figure at pixel 3.

Since the intensity of a CFI can be regarded as a two-dimensional (2D) sinusoidal signal, the background, modulation and phase distribution at pixel (*x*, *y*) thus can be demodulated by LEF. For example, as shown by the blue and red restrictions in Fig. 1(a), by plotting intensities of CFI at pixels [1 2 3 4 5] against that at pixels [2 3 4 5 6] a Lissajous figure can be created and subsequently fitted by EFA. The number here only denotes the index of the pixel, and a region of five pixels is the minimum requirement since at least five points are needed to fit an ellipse. We see the creating mode of Lissajous figure in Fig. 1(a) is one-dimensional (1D), therefore CFI with only one directional carrier is possible to demodulate using PLEF.

It is also easy to derive other means of producing the Lissajous figure at pixel (*x*, *y*). The one shown in Fig. 1(b) is a 2D mode which can be used for relatively lower carrier frequencies and higher noise levels. The first one, i.e. Figure 1(a) is used as an exemplary scheme in the following analysis of PLEF for the demodulation of CFI.

To demodulate CFI0 by PLEF, where CFI0 hereafter denotes a CFI that Eq. (2) represents for simplicity, the two sinusoidal signals of the Lissajous ellipse represented in Fig. 1(a) can be expressed in a more general way:

3 $${N}_{i}={A}_{i}+{B}_{i}\cdot \,\sin [{\varphi }_{i}],\,{D}_{i}={A^{\prime} }_{i}+{B^{\prime} }_{i}\cdot \,\cos [{\varphi }_{i}+{\rho }_{i}]\quad \quad i=1,\mathrm{2...5}$$

here subscript *i* denotes the index of the pixel (*x*, *y*), and

$${N}_{i}=I(x,i),$$

$${A}_{i}=A(x,i),$$

$${B}_{i}=B(x,i),$$

$${\varphi }_{i}=\phi (x,i)+\pi /2+{k}_{x}x+{k}_{y}i,$$

it is straightforward that

$${D}_{i}={N}_{i+1},$$

$$A{^{\prime} }_{i}={A}_{i+1},$$

$$B{^{\prime} }_{i}={B}_{i+1},$$

$${\rho }_{i}=\phi (x,\,i+1)-\phi (x,\,i)+{k}_{y}-\pi /2.$$

In the small region of CFI0, e.g. the green box shown in Fig. 1(a), if the background, modulation and phase of all pixels are constants, i.e.,

4 $${A}_{i}=A{^{\prime} }_{i}=A,\,{B}_{i}=B{^{\prime} }_{i}=B,\,{\rho }_{i}=\rho {^{\prime} }_{i}=\rho ,\quad \quad i=1,\mathrm{2...5}$$

where *ρ* = *k* _*y*_ − *π*/2. This is exactly the assumption of most SCPS methods. The exact expression of the ellipse, i.e. coefficients *A*, *B* and *ρ* that created from points (*N*_*i*_, *D*_*i*_) *i* = 1, 2...5 can be calculated^27^. Then the phase at pixel *i* can be demodulated correctly via the following equation:

5 $${\phi }_{i}={\tan }^{-1}\{1/[(\frac{{D}_{i}-A}{{N}_{i}-A})\frac{1}{\cos \,\rho }+\,\tan \,\rho ]\}-({k}_{x}\cdot x+{k}_{y}\cdot i+\frac{\pi }{2})$$

The final phase distribution can be demodulated by performing PLEF pixel by pixel over the entire CFI0. The demodulation process above, is represented as PLEF0.

The problem is, that in most cases, background *A*(*x*, *y*), modulation *B*(*x*, *y*) and *φ*(*x*, *y*) vary in a small region, that is Eq. (4) in CFI can be hardly satisfied in reality. The demodulated phase from Eq. (5) will, therefore, contain a detuning error caused by nonuniform illumination and local phase shift, which is also called local frequency or instantaneous frequency in some literatures^15^.

Though, background, modulation and phase may vary over five pixels, they are more likely to vary linearly in the small region^15^. Therefore, we can assume that variations of *A*(*x*, *y*), *B*(*x*, *y*) and *φ*(*x*, *y*) in a small region are constants,

6 $${dA(x,y)|}_{y=1,2,3,4,5}\,{=\delta A,dB(x,y)|}_{y=1,2,3,4,5}\,{=\delta B,d\phi (x,y)|}_{y=1,2,3,4,5}=\delta \phi $$

where,

*dA*(*x*, *y*) = *A*(*x*, *y* + 1) − *A*(*x*, *y*), *dB*(*x*, *y*) = *B*(*x*, *y* + 1) − *B*(*x*, *y*), *dφ*(*x*, *y*) = *φ*(*x*,*y* + 1) − *φ*(*x*, *y*).

Assuming the validity of Eq. (6), we can deduce that ${\rho }_{i}=\rho {^{\prime} }_{i}=\rho$ is still tenable in a small region of CFI0, nevertheless here *ρ* = *δφ* + *k* _y_− *π*/2 which indicates PLEF0 actually can compensate the local phase shift. However, nonuniform illumination namely variations in background and modulation are residual error sources that adversely affect the accuracy of PLEF0.

To address this problem, we propose to calculate the intensity difference between two adjacent pixels of CFI0. The intensity of an adjacent pixel, for example *I*(*x*, *y* + 1) is,

7 $$I(x,y+1)=A(x,y+1)+B(x,\,y+1)\cdot \,\cos \,[\phi (x,y+1)+{k}_{x}\cdot x+{k}_{y}\cdot (y+1)]$$

Subtracting two adjacent pixels, i.e. Eqs (2–7) yields

8 $$\overline{I}(x,\,y)=\alpha (x,\,y)+\beta (x,\,y)\cdot \,\sin [\phi (x,\,y)+{k}_{x}\cdot x+{k}_{y}\cdot y+{\rm{\Delta }}(x,\,y)/2]$$

where,

9 $$\alpha (x,\,y)=dA(x,\,y)+dB(x,\,y)\cdot \,\cos [{\rm{\Delta }}(x,\,y)/2]\cdot \,\cos [\phi (x,\,y)+{k}_{x}\cdot x+{k}_{y}\cdot y+{\rm{\Delta }}(x,\,y)/2]$$

10 $$\beta (x,\,y)=[B(x,\,y)+B(x,\,y+1)]\cdot \,\sin [{\rm{\Delta }}(x,\,y)/2]$$

11 $${\rm{\Delta }}(x,\,y)=d\phi (x,\,y)+{k}_{y}$$

Equation (8) represents another CFI, referred to here as CFI1, which is also encoded with the object phase. Eqs (9–11) denote the background, modulation and local phase shift of CFI1 respectively.

To demodulate CFI1, PLEF can be used in much the same way as PLEF0. We will denote the process as PLEF1. For CFI1 however, some coefficients in Eq. (3), now should be rewritten as

$${N}_{i}=\overline{I}(x,i),$$

$${A}_{i}=\alpha (x,i),$$

$${B}_{i}=\beta (x,i),$$

$${\varphi }_{i}=\phi (x,i)+{k}_{x}x+{k}_{y}i+{\rm{\Delta }}(x,i)/2,$$

$${\rho }_{i}={\rm{\Delta }}(x,\,y)-\pi /2$$

We find that in the small region of CFI1, with the validity of Eq. (6), variations of background, modulation and phase are,

12 $$d\alpha (x,\,y)=-dB(x,\,y)\cdot \,\sin [{\rm{\Delta }}(x,\,y)]\cdot \,\sin [\phi (x,\,y)+{k}_{x}\cdot x+{k}_{y}\cdot y+{\rm{\Delta }}(x,\,y)]$$

13 $$d\beta (x,\,y)=2dB(x,\,y)\cdot \,\sin [{\rm{\Delta }}(x,\,y)/2]$$

14 $$d{\rm{\Delta }}(x,\,y)=0$$

*dα*(*x*, *y*) and *dβ*(*x*, *y*) are very small values related both to the original variation of modulation, i.e. *dB*(*x*, *y*) and phase *φ*(*x*, *y*) but have no direct relation with the original background variation i.e. *dA*(*x*, *y*). Besides, local phase shift is still a constant in the small region of CFI1. That is to say, with the benefit of intensity difference process the original background variation is circumvented perfectly in CFI1. Whereas, in reality, the modulation may change significantly over the whole aperture, in this case the phase demodulation error is visible and therefore merits consideration.

With the calculated background *α′*(*x*, *y*) and modulation *β*′(*x*, *y*) by PLEF1, we propose to normalize CFI1, i.e.,

15 $$\overline{\overline{I}}(x,\,y)=\frac{\overline{I}(x,\,y)-\alpha ^{\prime} (x,\,y)}{\beta \text{'}(x,\,y)}$$

where *α*′(*x*, *y*) = *α*(*x*, *y*) + Δ_*α*_(*x*, *y*), *β′*(*x*, *y*) = *β*(*x*, *y*) + Δ_*β*_(*x*, *y*). Δ_*a*_(*x*, *y*) and Δ_*β*_(*x*, *y*) denote the demodulation error of background and modulation caused by the original modulation variation. Equation (16) represents a normalized CFI, referred to here as CFI2. Subsequently, PLEF is employed to demodulate CFI2 again (PLEF2). The final demodulation error caused by variation of modulation can be reduced effectively. The error compensation theory can be explained; the normalized intensity, i.e. CFI2 can be expressed as:

16 $$\overline{\overline{I}}(x,\,y)=\frac{{{\rm{\Delta }}}_{\alpha }(x,\,y)}{\beta \text{'}(x,\,y)}+\frac{1}{1+{{\rm{\Delta }}}_{\beta }(x,\,y)/\beta ^{\prime} (x,\,y)}\cdot \,\sin [\phi (x,\,y)+{k}_{x}\cdot x+{k}_{y}\cdot y+\frac{1}{2}{\rm{\Delta }}(x,\,y)]$$

Although the exact expressions of Δ_*a*_(*x*, *y*) and Δ_*β*_(*x*, *y*) are unknown, there is no doubt that both $\frac{{{\rm{\Delta }}}_{\alpha }(x,\,y)}{\beta ^{\prime} (x,\,y)}$ and $\frac{{{\rm{\Delta }}}_{\beta }(x,\,y)}{\beta ^{\prime} (x,\,y)}$ are negligible. In the normalized fringe pattern, illuminations of the adjacent five pixels are approximately uniform, which ensures ultimate high accuracy phase demodulation.

**Figure 2:**
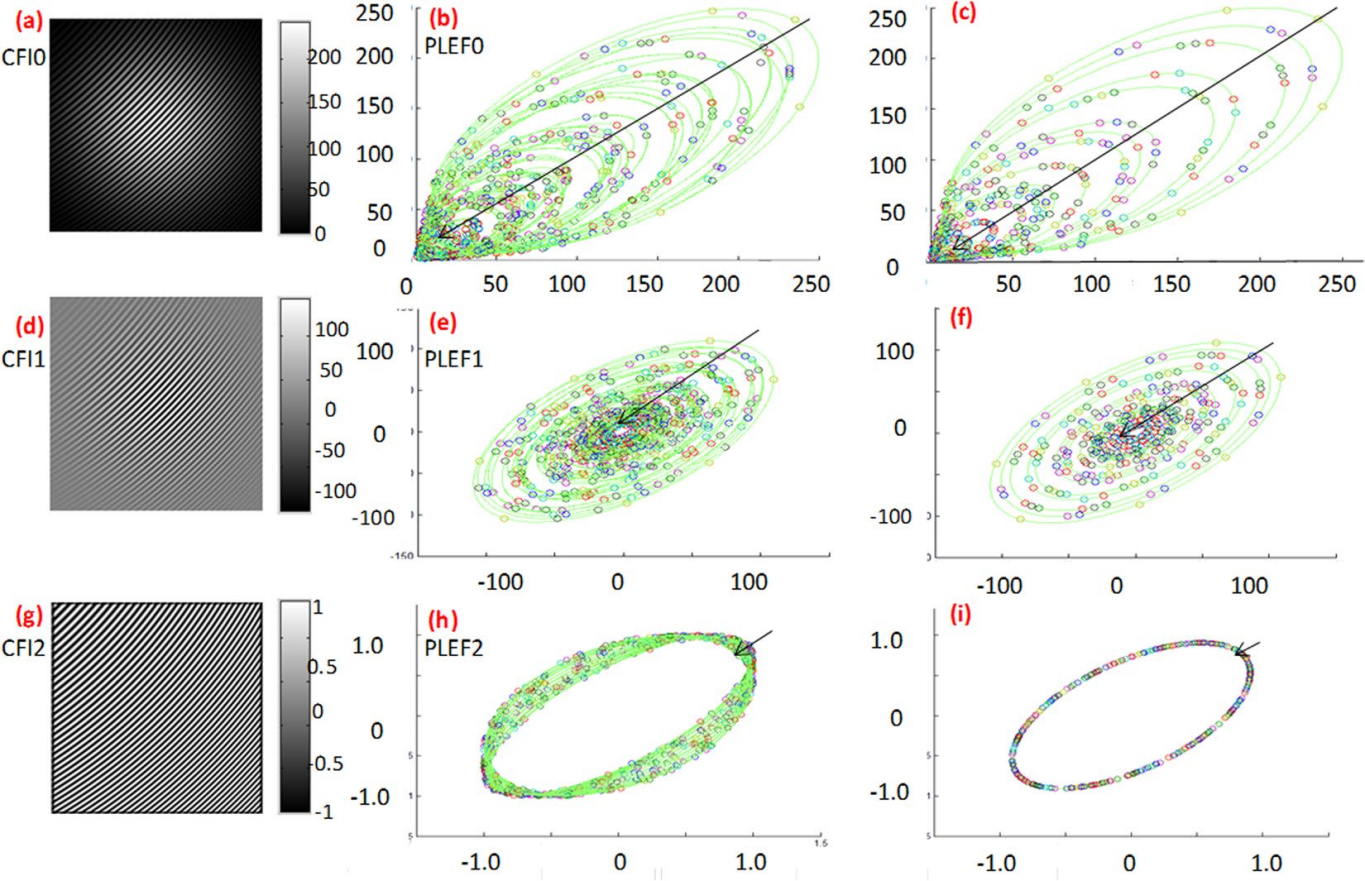
The demodulation procedure of PLEF: the first row represents (**a**) CFI0 with significant variations in background and modulation, (**b**) the real Lissajous ellipses at all pixels in (**a**), (**c**) the reference Lissajous ellipses at all pixels in (**a**); similarly the second row and the third row represent the (**d**) CFI1, (**e**) the real Lissajous ellipses at all pixels in (**d**), (**f**) the reference Lissajous ellipses at all pixels in (**d**); (**g**) normalized CFI2, (**h**) the real Lissajous ellipses at all pixels in (**g**), (**i**) the reference Lissajous ellipses at all pixels in (**g**).

The implementation of proposed method is illustrated in Fig. 2. If both illumination and the encoded phase are changed significantly, the CFI0 is as shown in Fig. 2(a). At each pixel of CFI0, a Lissajous figure can be created directly. Figure 2(b) demonstrates the Lissajous ellipses that created at all pixels in CFI0. Error-free Lissajous ellipses (created by pixels with uniform illumination) are correspondingly plotted in Fig. 2(c). On comparison of Fig. 2(b) with Fig. 2(c), it becomes apparent that the ellipse is more likely to be error free at the center of CFI0 where there are only slight variations in illumination and phase, whereas the error increases dramatically at the CFI0 border. Note that we assume the illumination obeys Gaussian distribution. PLEF0 is affected by distinct detuning error caused by background/modulation variation. If the intensity difference of CFI0, i.e. CFI1, as shown in Fig. 2(d) is calculated, then the Lissajous ellipses at all pixels shown in Fig. 2(e) are all centered around zero since the original background is deducted. Errors still exist compared with the corresponding error-free ellipses, i.e. Fig. 2(f), which is caused mainly by modulation variation. Figure 2(g) represents normalized CFI2 which has approximately zero background and unit modulation. In this case the error-free ellipses at all pixels should be overlapped like the one shown in Fig. 2(i). The quadrature phase shift error represents the carrier frequency. Because the actual local phase shift of each pixel differs, the corresponding Lissajous ellipses will not be completely overlapped as shown in Fig. 2(i). They bear a closer resemblance to those in Fig. 2(h), which exactly demonstrates the proposed PLEF has the ability of local phase shift compensation. We can see that axes in Fig. 2 are different which is straightforward since CFI0, CFI1 and CFI2 have dissimilar Intensities.

## Results

A simulation followed by a quasi-experiment has been conducted in order to verify the correctness of the proposed method and scrutinize its performance. The CFI is synchronized according to Eq. (1) here

$$A(x,\,y)=130\exp [{r}_{a}({x}^{2}+{y}^{2})],B(x,\,y)=120\exp [{r}_{b}({x}^{2}+{y}^{2})]$$

where −1 ≤ *x*, *y* ≤ 1 and *r*_*a*_, *r*_*b*_ determines the variation amplitude of the background and modulation over the entire CFI. ILSM^19^ can effectively compensate variations of background and modulation as well as the local phase shift error. It is, therefore always regarded as a reference when comparing the performance of SCPS methods^20,22^. In this simulation, most parameters in Xu’s simulation^19^ are purposely inherent to facilitate a straightforward comparison, thereby avoiding the personal ILSM error. Without loss of generality, the phase size is 200 × 200 and the object phase is also set to *φ*(*x*, *y*) = 2*π*(*x* ^2^ + *y* ^2^). *r*_*a*_ = *r*_*b*_ = 0.1 and phase carrier is *k*_*x*_ = *k*_*y*_ = 1.24 rad/pixel. Additive noise here is zero for the convenience of theoretical error analysis.

**Figure 3:**
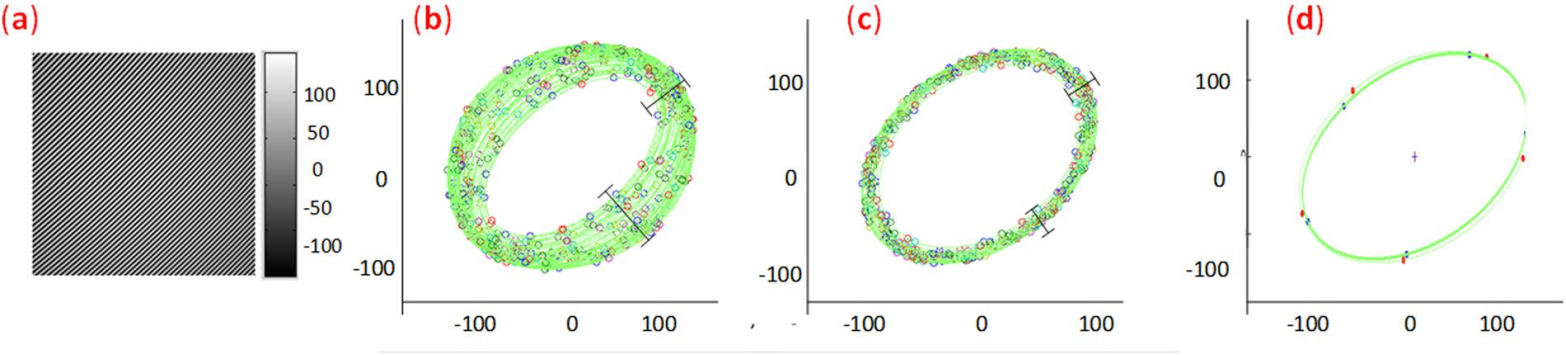
(**a**) CFI1, (**b**) The Lissajous ellipses at each pixel, (**c**) the corresponding error free ones, (**d**) the comparison of the actual Lissajous ellipse and reference Lissajous ellipse at pixel (80, 80).

**Figure 4:**
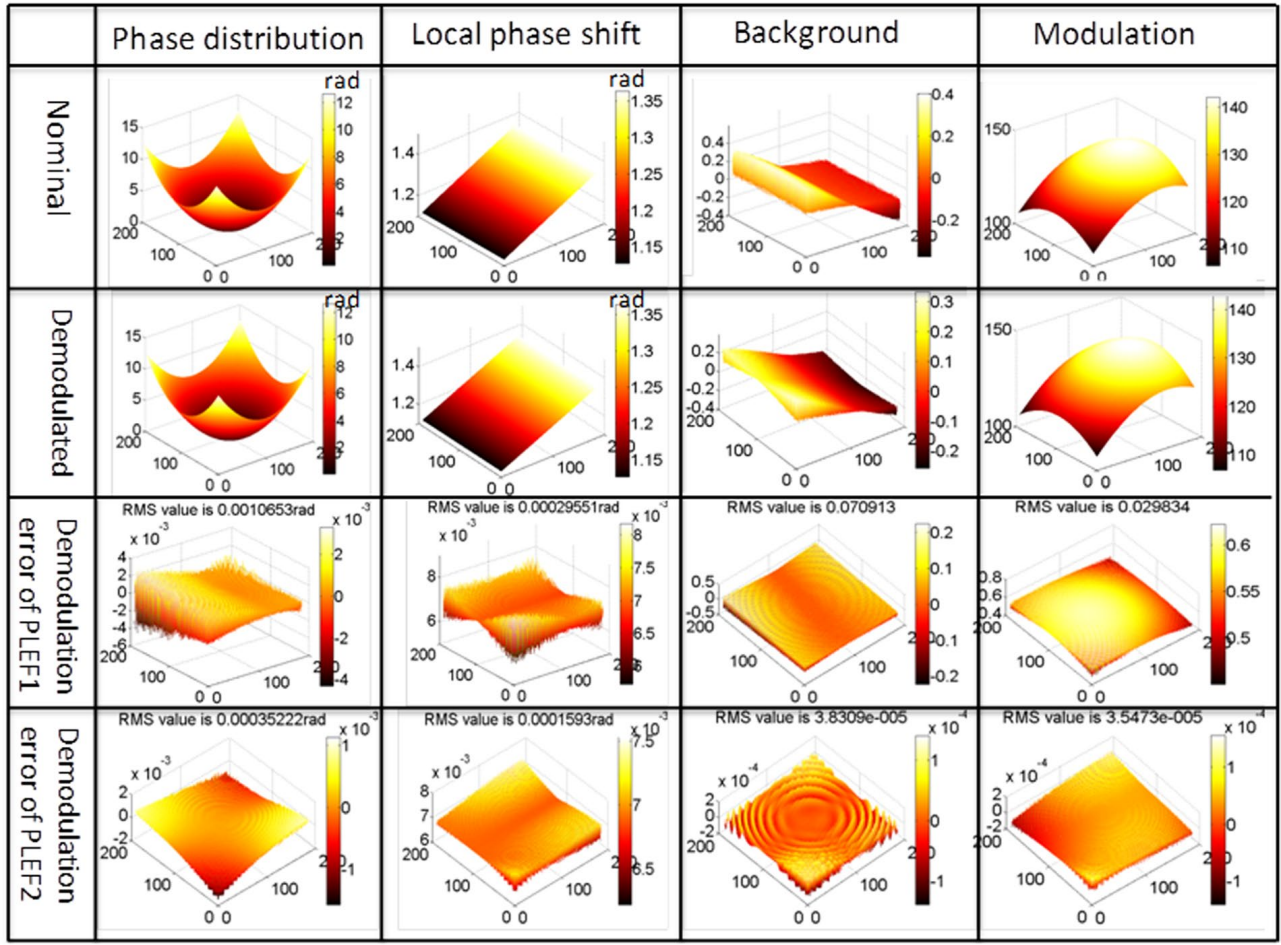
Demonstration of the correctness and theoretical error of the proposed PLEF.

The CFI is synchronized with the assigned parameters. First, the intensity difference of the fringe pattern, i.e. CFI1, shown in Fig. 3(a), is calculated. Figure 3(b) shows the Lissajous ellipses created at each pixel of CFI1. The corresponding error-free ellipses of Fig. 3(b) are also created and shown in Fig. 3(c) in order to demonstrate theoretical error. As an example, the Lissajous ellipse created at pixel (80, 80) with real dots (blue), and the error-free ellipse, created with reference dots(red) are simultaneously plotted in Fig. 3(d). Clearly, the real dots (blue) deviate considerably from the reference dots (red), thus the fitted ellipse is both decentered (background) and biased (modulation) compared with the reference ellipse. That is the theoretical error of the proposed method. In order to quantify the theoretical error of PLEF, phase distribution, background, modulation and local phase shift of CFI1 are demodulated by PLEF1 and the corresponding residual error maps are also calculated by comparing with the nominal values according to the mathematic expressions, i.e. Eqs (9–11). Figure 4 represents the demodulation results and corresponding residual error maps of the method proposed. The ultimate phase demodulation error of PLEF2 can be further reduced to 3.5 × 10^−4^ rad (RMS) even though the background/modulation is highly non-uniform and the maximum local phase gradient is higher than 0.11 rad.

**Figure 5:**
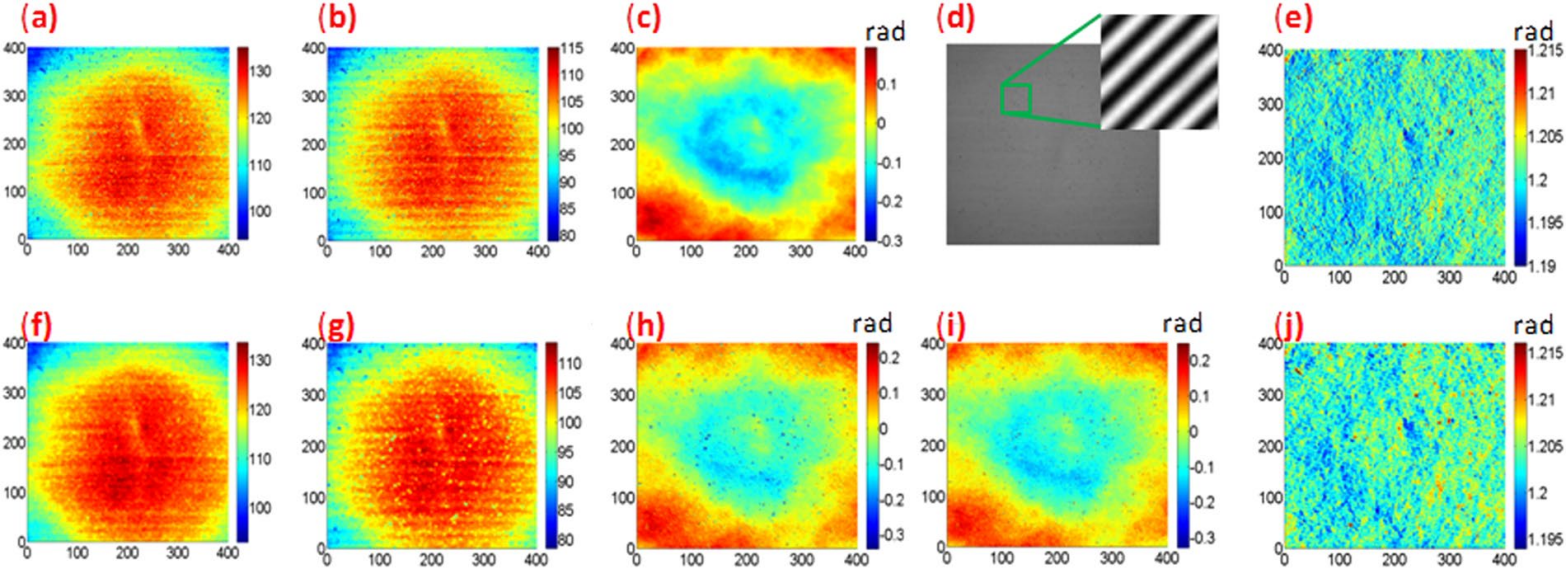
The experimental application of the proposed PLEF: calculated (**a**) background, (**b**) modulation amplitude, (**c**) the real phase measured by a Zygo interferometer; (**d**) synchronized quasi-practical CFI with calculated real background, modulation and real phase, (**e**) the reference local phase shift; the retrieved (**f**) background, (**g**) modulation amplitude, (**j**) local phase shift, (**h**) phase distribution by PLEF0; (**i**) the extracted phase by PLEF1.

To further demonstrate the validity of the proposed method when background, modulation and phase variation are all small, the following experiment was conducted. An optical flat was first measured at null condition using a Zygo GPI interferometer. With the saved interferograms (100 frames, each frame containing only two fringes), the background and modulation of these interferograms were calculated using the maxim and minum method^30^. These are shown in Figs. 5(a),(b) measuring 400 × 400. A quasi practical CFI, shown in Fig. 5(d) is produced according to Eq. (1) with the calculated background intensity, modulation amplitude and real phase measured by the Zygo interferometer at null condition which is shown in Fig. 5(c). This permitted the carrier frequency introduced to be controlled without introducing a retrace error, here *k*_*x*_ = *k*_*y*_ = 1.20 rad/pixel. The local phase shift along the *y* direction of Fig. 5(c) is shown in Fig. 5(e), which demonstrates that the phase variation is very low (PV < 0.02 rad). The proposed PLEF0 method is first executed in order to demodulate CFI0. The demodulated background, modulation and final phase distribution are shown in Figs. 5(f–h) respectively. Figure 5(j) shows the local phase shift. Compared with the nominal ones, PLEF0 can already demodulate the CFI correctly. The residual phase error is 0.035 rad (RMS), which is mainly background noise. PLEF1 was also used to demodulate the phase, the phase is shown in Fig. 5(i). As shown, there’s a slight improvement in the quality of the retrieved phase and the phase residual error is 0.031 rad in RMS. It demonstrates that PLEF0 can be used when the illumination variation is small, e.g. less than 30% in reality, whereas PLEF1 or even PLEF2 can still be used to improve the performance of PLEF.

## Discussion

Although the correctness of the mathematical model and the high accuracy of the proposed PLEF method were demonstrated in the previous simulation, theoretical errors remain. As previously outlined^20,22,23^, the background, modulation, encoded phase, phase carrier, fringe tilt angle and additive noise can all affect the accuracy of SCPS algorithms. Yet the proposed method performs well. Next the validity of the proposed method in a harsh condition will be demonstrated and followed by a comprehensive discussion focusing on different influencing factors. The encoded phase is created by the *peaks* function in Matlab. The variation parameters of background and modulation are set to *r*_*a*_ = *r*_*b*_ = 0.8, which indicate the illumination amplitude at the edge is only 20.1% of that in the center. The carrier frequencies are separately set to *k*_*x*_ = 0, *k*_*y*_ = 1.24 rad/pixel, which means the tilt angle of the fringe is zero. Additionally, a Gaussian white noise with the SNR of 30 dB is added by the *awgn* function using the “*measured*” option in Matlab, the maximum noise gray level is about 15.

**Figure 6:**
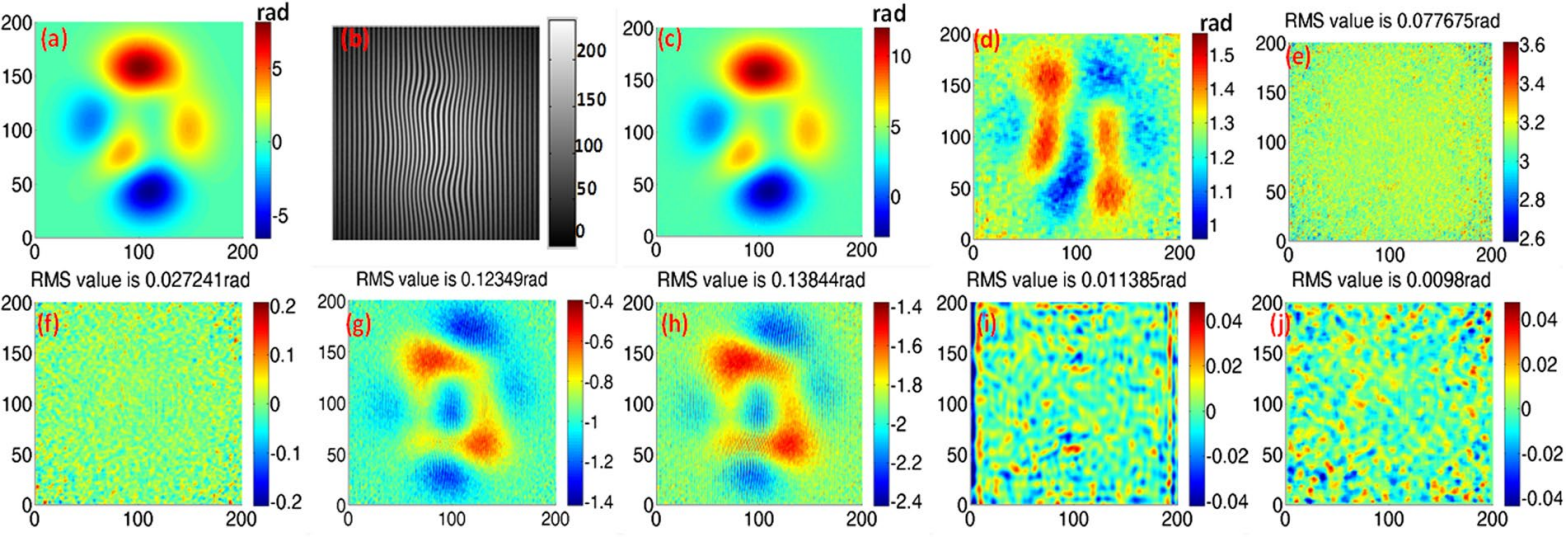
The effectiveness of proposed PLEF and comparison with AIA\PCA based SCPS methods and WFT based WFF: (**a**) object peaks phase, (**b**) CFI0; demodulated (**c**) phase and (**d**) local phase shift by PLEF1; demodulation error map of (**e**) PLEF1, (**f**) PLEF2, (**g**) AIA, (**h**) PCA, (**i**)WFF; (**j**) PLEF2 filtered by medfilt2.

Figure 6(a) is the object phase and Fig. 6(b) denotes the original synchronized interferogram CFI0. PLEF1 is directly executed for the dramatic variation of background. The demodulated phase distribution and local phase shift are shown in Fig. 6(c,d). The phase demodulation error map of PLEF1 is shown in Fig. 6(e) with the RMS of 0.078 rad, from which we can see the high frequency noise is the main component of residual error. Therefore, a Gaussian low pass filter is utilized before PLEF2 is executed to reduce the effect of noise. Figure 6(f) displays the subsequent residual error map of PLEF2 with the RMS value of 0.027 rad.

In order to facilitate comparison, AIA and PCA based SCPS methods are also employed to perform the demodulation. The residual phase error maps are shown in Fig. 6(g,h). Note that since the tilt angle is zero, 9 phase shifted interferograms need to be decomposed from CFI0 in both AIA and PCA based methods to avoid under-sampling^20^. The residual errors are 0.123 rad (RMS) and 0.138 rad (RMS) respectively and are mainly phase-dependent errors for less of local phase shift compensation.

As a local process, WFT has been recognized as a powerful mathematic tool for fringe patterns analysis^15^. Thus, the WFT based windowed Fourier Filtering (WFF) is selected for comparison in this simulation. According to the suggested frequency ranges in ref.^15^, the parameters are listed in the first row of Table 1, where *σ*, *ξ*_*l*_, *ξ*_*h*_, *η*_*l*_, *η*_*h*_ respectively denotes window size, low band of frequency in *x*, high band of frequency in *x*, low band of frequency in *y*, high band of frequency in *y*. The demodulation error of WFF is represented by *e*. Table 1 indicates that, it is typically difficult to select the proper frequency ranges in WFF since we absolutely don’t know the carrier frequency of CFI previously. By attempting different ranges of frequency and window sizes, we approximate the parameters lead to the minimum phase demodulation error. The residual error map is shown in Fig. 6(i), it is obvious WFF can successfully compensate the local phase shift error. Though the RMS value of Fig. 6(i) is only 0.0114 rad and is smaller than proposed PLEF2, high frequency information is missing compared with Fig. 6(f). Actually, if a *medfilt2* filter with window size of 7 × 7 is utilized for Fig. 6(f), residual phase error map of PLEF2 with a even less RMS value will shows like Fig. 6(j). Besides, Fig. 6(i) indicates the edge error of WFF is obvious which is unavoidable because of the inherent characteristic of FT based methods.

**Table 1:** Selection of proper frequency range.

index parameters	**σ**	***ξ***_**1**_	***ξ***_***h***_	***η***_**1**_	***η***_***h***_	***e*** **(rad)**
1	10	−0.2	0.2	1	2.1	0.068
2	10	−0.5	0.5	1	2.1	0.019
3	10	−1	1	1	2.1	0.020
4	10	−1	1	0.8	2.1	0.012
5	10	−1	1	0.6	2.1	0.012
6	4	−1	1	0.6	2.1	0.014

**Figure 7:**
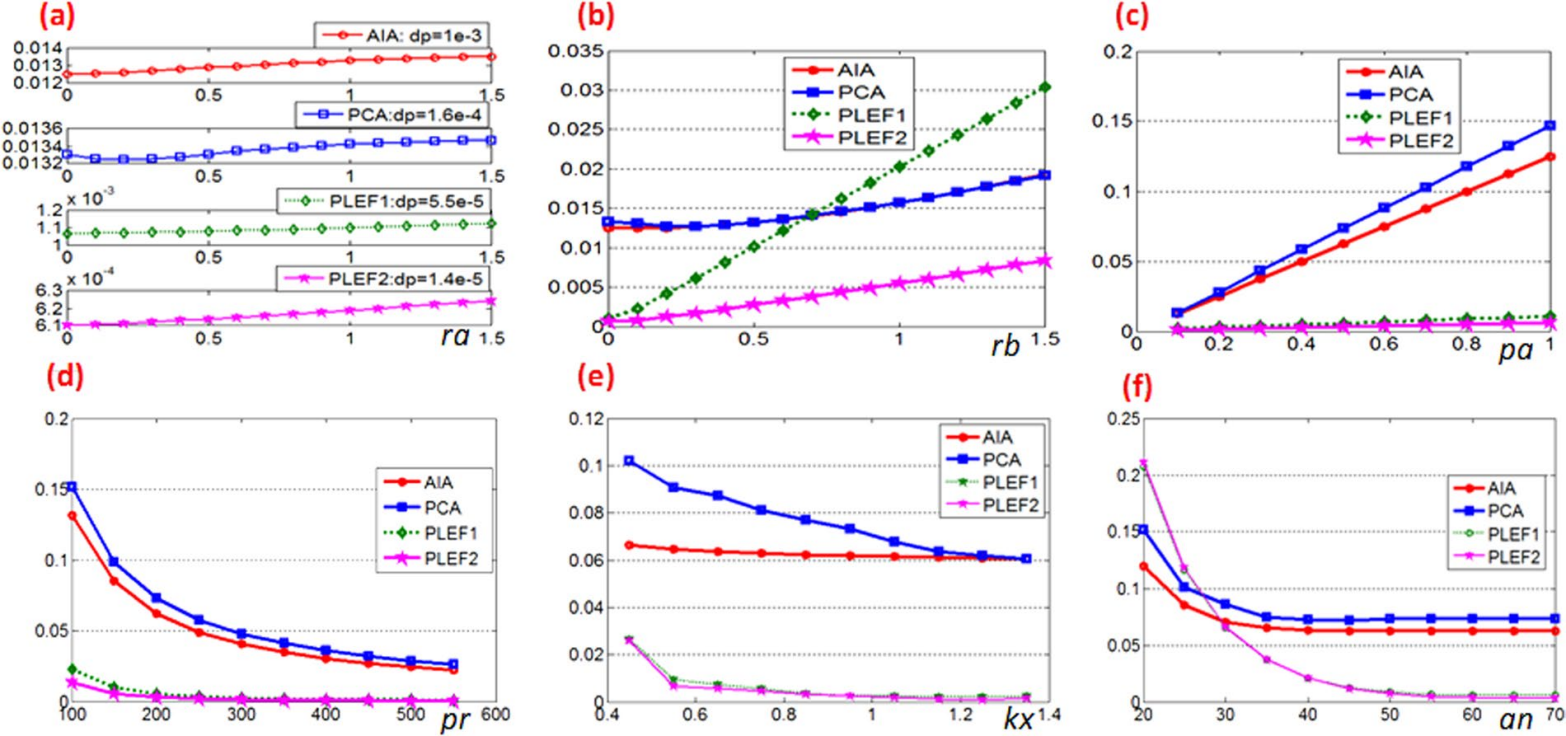
The performances of AIA, PCA based SCPS algorithms and proposed PLEF versus various influencing factors (**a**) background, (**b**) modulation, (**c**) phase amplitude, (**d**) resolution, (**e**) carrier frequency, (**f**) additive noise level.

Different parameter settings will lead directly to different results and different algorithms typically have divergent responses. The performance of the proposed PLEF algorithm under a diverse range of influencing factors is discussed in detail in the following. The main advantages of the method proposed in comparison with AIA and PCA based SCPS algorithms are illustrated using graphs. Here, WFF is out of comparison since proper frequency ranges can not be selected automatically. Influences exerted by background variation *ra*, modulation variation *rb*, phase amplitude *pa*, phase size *pr*, phase carrier frequency *kx, ky*, and additive noise *an*, will be discussed individually. In the following Fig. 7, each horizontal axis denotes a different influencing factor, however all vertical axes represent the phase demodulation error (evaluated by RMS value, the unit is rad).

First, the influence exerted by the background was analyzed. The range of *ra* is 0 to 1.5 with the interval of 0.1, *rb* = 0.01, *pa* = 0.1, *pr* = 200, *kx* = 0, *ky* = 1.24 and no additional noise. Please note that other factors were purposely set to small in order to highlight background influence. Figure 7(a) indicates that with the increase of *ra* from 0 to 1.5, PLEF2 has the smallest phase demodulation variation (dp) is only 1.4 × 10^−5^ rad. AIA is the most sensitive to background variation because of non-compensation of background.

Secondly, the influence exerted by modulation was investigated. The value *rb* was allowed to vary from 0 to 1.5. Similar to the above described analysis, *ra* = 0.01 and other parameters stayed the same. Figure 7(b) shows that though PLEF1 is very sensitive to variation in modulation, PLEF2, whose response to modulation variation is similar to that of AIA and PCA, can significantly reduce the impact of modulation variation.

As demonstrated in Figs. 7(a) and (b), the benefit of the local phase shift compensation in the method proposed, is the increase in accuracy (the RMS value of the phase demodulation error is almost one magnitude smaller) than AIA and PCA-based SCPS methods even at a high illumination variation level. This can be further underlined with Fig. 7(c,d).

In Fig. 7(c), the phase size is fixed to 200 × 200, i.e. *pr* = 200. The phase amplitude, *pa*, varies from 0.1 to 1 with the interval of 0.1, *ra* = *rb* = 0.1, *kx* = 0, *ky* = 1.24. No account is taken of noise. In Fig. 7(d), the phase amplitude *pa* is fixed to 0.5, the phase size, *pr*, varies from 100 to 550 with an interval of 50; other parameters stay the same as in Fig. 7(c). Figure 7(c) indicates that with increasing of phase amplitude (local phase gradient increases) the theoretical errors of AIA/PCA based methods undergo dramatic linear increases whereas the increase in the PLEF outlined here is much more gradual. Similarly, Fig. 7(d) shows that with increasing of phase size (local phase gradient decreases), the theoretical error decreases regardless of the method applied. The distinct difference between AIA/PCA based methods and the PLEF method proposed in Figs. 7(c),(d) demonstrates powerfully that the proposed PLEF can effectively compensate the local phase shift error whereas traditional SCPS methods cannot.

Subsequently, the phase carrier-frequency is studied. Since AIA and PCA based SCPS methods behave better when the tilt angle is 45 deg^19,20^, here it is set to 45 deg but it is important to bear in mind that as previously demonstrated, the proposed PLEF is insensitive to tilt angle. We let *kx* = *ky* vary from 0.45 rad/pixel to 1.35 rad/pixel, *ra* = *rb* = 0.1, *pa* = 0.5 without noise. Disregarding the advantages of local phase shift compensation, it is apparent that the response of both AIA-based SCPS and the proposed PLEF to carrier frequency is more stable than that of PCA, as shown in Fig. 7(e), while PLEF1 and PLEF2 furthermore result in significant lower phase demodulation errors compared to AIA-based SCPS. Since only five consecutive points are used to create the Lissajous figure in the proposed PLEF, it is better when the phase carrier between adjacent pixels is higher than 1.2 (2*π*/5) rad, which ensures that the Lissajous figure is closed. However, as outlined in ref.^29^, the ellipse fitting can also be complemented successfully and accurately even though the Lissajous figure is open. In this simulation we find that if the phase carrier-frequency exceeds 0.4 rad/pixel, which is higher than a quarter of an ellipse, the proposed method is effective.

Finally, additive noise is considered. The SNR of the noise varies from 20 dB to 70 dB, *ra* = *rb* = 0.1, *pa* = 0.5, *kx* = 0 and *ky* = 1.24 rad/pixel. The comparison reveals that the proposed method is much more sensitive to additive noise than AIA and PCA-based SCPSs, because PLEF is a local demodulation method and the Lissajous figure is created using only five adjacent points rendering it more sensitive than the phase shifting method. By selecting more points, i.e. enlarging the size of the window to create the Lissajous figure, e.g. Fig. 1(b), it is possible to enhance the stability of the proposed method to noise. Additionally, the averaging *N* measurements can effectively reduce the residual error by $\sqrt{N}$ times to the theoretical error limit. As shown in Fig. 6(f), an image denoising process can also significantly improve the accuracy of the proposed method. PLEF will, therefore, ultimately achieve high accuracy whereas AIA and PCA-based SCPS methods cannot since their theoretical errors are mainly phase-dependent.

From Fig. 7 one may notice that the phase demodulation error that caused by variation of background or modulation is very small comparing to other influencing factors, e.g. noise level. That’s why PLEF1 and PLEF2 in Figs. 7(c–f) have almost the same response to different influencing factors. In practice therefore, if variation of illumination is less than 30% PLEF0 is accurate enough as Fig. 5(h) already demonstrated. However, in case of illumination has a considerable variation, PLEF1 or even PLEF2 is recommended to achieve a higher precision of phase demodulation if the fringe can be denoised in advance.

**Figure 8:**
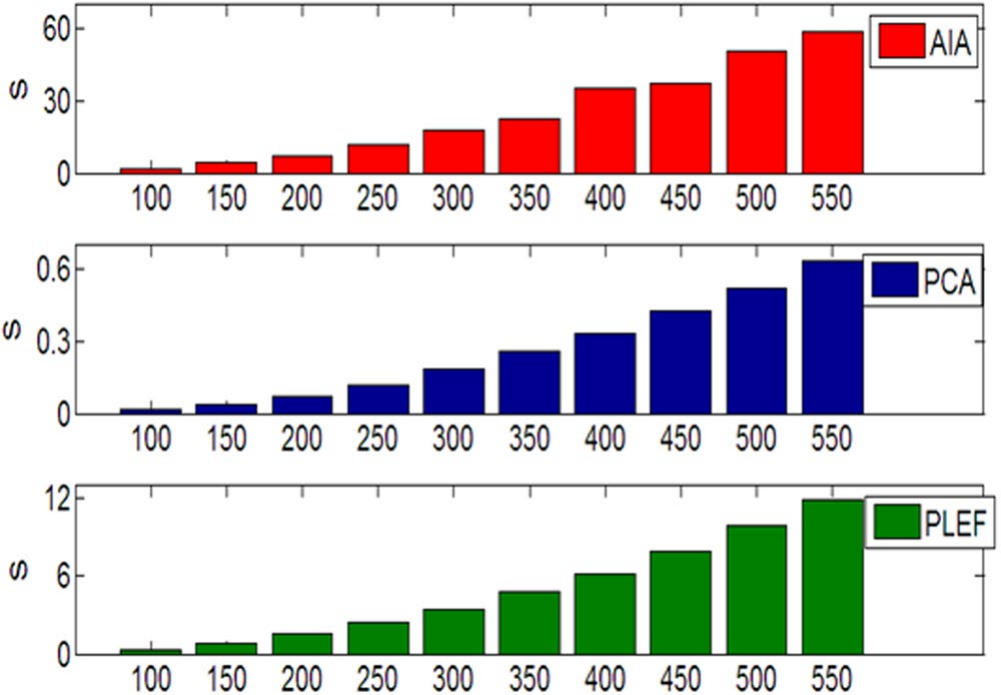
The computing time of AIA, PCA-based SCPS algorithms and the proposed PLEF versus different phase size.

In addition to phase-demodulation accuracy, computing speed is also of interest in terms of dynamic measurement. The processing time required by AIA, PCA-based SCPS methods and PLEF are calculated when the influence exerted by phase size is analyzed. The computing is executed by an Intel i5 CPU with 3.2 GHz frequency in Matlab. The bar in Fig. 8 indicates that PCA- based SCPS is the least time-consuming among the three algorithms, for CFI with size of 400 × 400 it cost only 0.42 s whereas AIA based SCPS needs 36.3 s which is typically two magnitudes slower than PCA. For proposed PLEF, it costs 6.2 s which is also time consuming than PCA because each pixel requires an ellipse fitting process, nevertheless it is typically 5~6 times faster than AIA without the convergence problem. The PLEF method proposed here is similar with WFT method, data parallelism is possible. With the help of a multi core CPU or GPU platform, high accuracy dynamic demodulation can be achieved.

## Conclusions

In this paper, we have presented a pixel-level Lissajous figure and ellipse-fitting (PLEF) method to demodulate the phase distribution from a single-phase carrier fringe pattern. The flexibility and accuracy achieved are higher than the levels achieved using the FT method. With a more generic assumption, the intrinsic character of the Lissajous figure makes it more tolerant to the local phase shift caused by the phase gradient. Variations in background and modulation are two further error sources for SCPS. The phase demodulation errors thereby caused, can be reduced separately by subtracting adjacent pixels (PLEF1) and by normalizing the fringe with the calculated background and modulation (PLEF2). Greater accuracy can be achieved within an acceptable timeframe using the method outlined in this paper than that expected using AIA and PCA-based SCPS methods. It should, therefore, be a better candidate for single-phase carrier fringe pattern analysis in terms of both accuracy and speed.

## References

[1] Malacara, D., Servín, M. & Malacara, Z. Interferogram analysis for optical testing (Vol. 84, CRC, 2005).

[2] Deck LL (2009). Suppressing phase errors from vibration in phase-shifting interferometry. Appl Optics.

[3] Cheng YY, Wyant JC (1985). Phase shifter calibration in phase-shifting interferometry. Appl Optics.

[4] Surrel Y (1993). Phase stepping: a new self-calibrating algorithm. Appl Optics.

[5] Greivenkamp JE (1984). Generalized Data Reduction for Heterodyne Interferometry. Opt Eng.

[6] Okada K, Sato A, Tsujiuchi J (1991). Simultaneous calculation of phase distribution and scanning phase shift in phase shifting interferometry. Opt Commun.

[7] Wang Z, Han B (2004). Advanced iterative algorithm for phase extraction of randomly phase-shifted interferograms. Opt Lett.

[8] Juarez-Salazar R, Robledo-Sanchez C, Meneses-Fabian C, Guerrero-Sánchez F, Aguilar LA (2013). Generalized phase-shifting interferometry by parameter estimation with the least squares method. Opt Laser Eng.

[9] Liu F, Wu Y, Wu F, Song W (2016). Generalized phase shifting interferometry based on Lissajous calibration technology. Opt Laser Eng.

[10] Millerd, J. E., Brock, N. J., Baer, J. W. & Spuhler, P. Vibration insensitive, interferometric measurements of mirror surface figures under cryogenic conditions, *Proc. SPIE*, 4842–41 (2002)

[11] Sun P, Zhong L, Luo C, Niu W, Lu X (2015). Visual measurement of the evaporation process of a sessile droplet by dual-channel simultaneous phase-shifting interferometry. Sci Rep.

[12] Koliopoulos CL (1992). Simultaneous phase-shift interferometer. Proc. SPIE.

[13] Takeda M, Ina H, Kobayashi S (1982). Fourier-transform method of fringe-pattern analysis for computer-based topography and interferometry. JOSA.

[14] Takeda M (1990). Spatial-carrier fringe-pattern analysis and its applications to precision interferometry and profilometry: an overview. Industrial Metrology.

[15] Kemao Q (2007). Two-dimensional windowed Fourier transform for fringe pattern analysis: principles, applications and implementations. Opt Laser Eng.

[16] Kujawinska M, Wojciak J (1991). Spatial-carrier phase-shifting technique of fringe pattern analysis. Proc. SPIE.

[17] Debnath SK, Park Y (2011). Real-time quantitative phase imaging with a spatial phase-shifting algorithm. Opt Lett.

[18] Vargas J, Quiroga JA, Belenguer T (2011). Phase-shifting interferometry based on principal component analysis. Opt Lett.

[19] Xu J, Xu Q, Peng H (2008). Spatial carrier phase-shifting algorithm based on least-squares iteration. Appl Optics.

[20] Du Y, Feng G, Li H, Vargas J, Zhou S (2012). Spatial carrier phase-shifting algorithm based on principal component analysis method. Opt Express.

[21] Zhang R, Guo H (2014). Phase gradients from intensity gradients: a method of spatial carrier fringe pattern analysis. Opt Express.

[22] Huang L (2016). Dynamic phase measurement based on spatial carrier-frequency phase-shifting method. Opt Express.

[23] Xu J, Jing W, Cai L, Xu Q (2011). Phase extraction from randomly phase-shifted interferograms by combining principal component analysis and least squares method. Opt. Express.

[24] Vargas J, Quiroga JA, Belenguer T (2011). Analysis of the principal component algorithm in phase-shifting interferometry. Opt Lett.

[25] Vargas J, Sorzano COS, Estrada JC, Carazo JM (2013). Generalization of the Principal Component Analysis algorithm for interferometry. Opt Communications.

[26] Vargas J, Carazo JM, Sorzano COS (2014). Error analysis of the principal component analysis demodulation algorithm. Appl Physics B.

[27] Liu F, Wu Y, Wu F (2015). Correction of phase extraction error in phase-shifting interferometry based on Lissajous figure and ellipse fitting technology. Opt Express.

[28] Farrell CT, Player MA (1992). Phase-step measurement and variable step algorithms in phase-shifting interferometry. Meas. Sci. Technol..

[29] Liu F (2016). Simultaneous extraction of phase and phase shift from two interferograms using Lissajous figure and ellipse fitting technology with Hilbert–Huang prefiltering. Journal of Optics.

[30] Liu F, Wu Y, Wu F (2015). Phase shifting interferometry from two normalized interferograms with random tilt phase-shift. Opt Express.

